# Supplementary material for: Immunodeficient mice are better for modeling the transfusion of human blood components than wild-type mice
Source: PLoS One. 2020 Jul 31;15(7):e0237106. doi: 10.1371/journal.pone.0237106 (PMC7394438; doi:10.1371/journal.pone.0237106)
Supplement: S1 File — (PDF) [file pone.0237106.s001.pdf]

Supplemental Tables of Statistical Analyses and Descriptive Statistics for Fig. 1E

S1 Table. P-values for CD235a+ erythrocytes for Fig 1E.*

	B6	BALB/c	FVBN
	**5 Minutes**		
NSG	0.0122	0.0090	0.0758
B6		0.0141	0.1172
BALB/c			0.0163
	**2 Hours**		
NSG	0.0090	0.0090	0.1172
B6		0.0090	0.0472
BALB/c			0.0090
	**20 Hours**		
NSG	0.0090	0.0090	0.0090
B6		0.1172	0.0090
BALB/c			0.0090

S2 Table. P-values for CD45+ leukocytes for Fig 1E.*

	B6	BALB/c	FVBN
	**5 Minutes**		
NSG	0.0122	0.0090	0.0090
B6		0.0090	0.2506
BALB/c			0.0472
	**2 Hours**		
NSG	0.0090	0.0090	0.2506
B6		0.4647	0.0472
BALB/c			0.0163
	**20 Hours**		
NSG	0.0090	0.0090	0.0090
B6		0.0090	0.0472
BALB/c			0.9168

S3 Table: P-values for CD41+CD42b+ platelets for Fig 1E.*

	B6	BALB/c	FVBN
	**5 Minutes**		
NSG	0.0216	0.0090	0.0090
B6		0.0090	0.0758
BALB/c			0.0758
	**2 Hours**		
NSG	0.0090	0.0090	0.0090
B6		0.3472	0.0758
BALB/c			0.0163
	**20 Hours**		
NSG	0.0090	0.0090	0.0090
B6		0.6015	0.9168
BALB/c			0.6015

S4 Table: Recipient weights in grams for Fig 1E.

	Mean	Median	Minimum	Maximum	N
NSG	22.68	22.90	20.3	25.3	5
B6	19.50	19.60	18.4	20.7	5
BALB/c	21.70	21.20	20.8	23.9	5
FVBN	22.86	22.90	21.5	23.6	5

*All P-values were obtained by unpaired, two-tailed, comparisons made using the Mann-Whitney U-test.
