# Supplementary material for: Immunodeficient mice are better for modeling the transfusion of human blood components than wild-type mice
Source: PLoS One. 2020 Jul 31;15(7):e0237106. doi: 10.1371/journal.pone.0237106 (PMC7394438; doi:10.1371/journal.pone.0237106)
Supplement: S1 Fig — The indicated strains of mice were transfused with whole blood containing 1.86 x 10^6^ leukocytes, 6.10 x 10^8^ erythrocytes, and 5.54 x 10^7^ platelets. (A) Cytokines detected in the sera that were unaffected by transfusion are shown. Cytokines for which only some of the highest measurements were in range of the standard curve are shown in (B) and cytokines whose concentration fell bellow accurate detection levels are shown in (C). Significant differences between pre- and post-transfusion are indicated by an asterisk by the name of the mouse strain. (PDF) [file pone.0237106.s002.pdf]

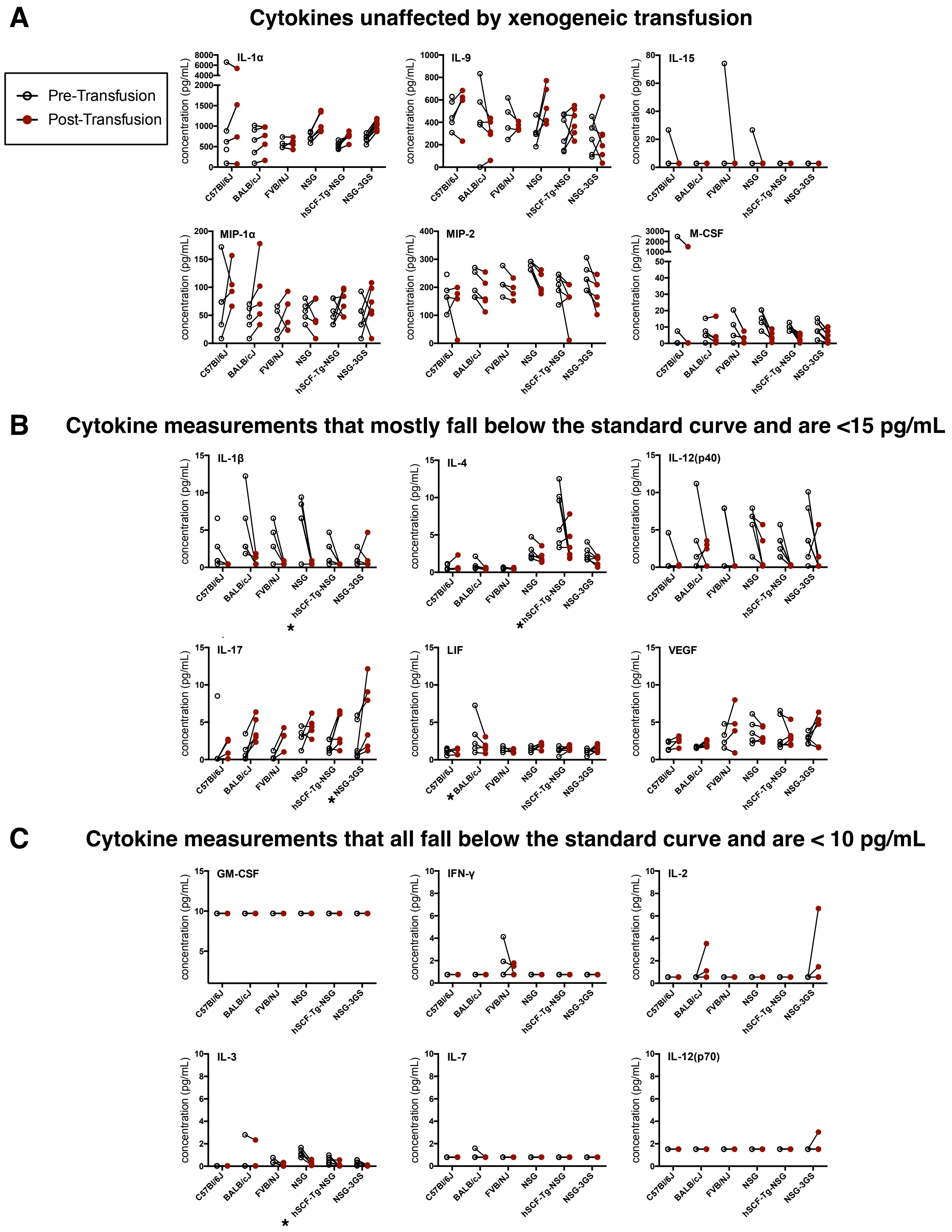

S1 Figure. Cytokine levels in the serum of mice before and 1.5 hours after transfusion with human blood. The indicated strains of mice were transfused with whole blood containing 1.86 x 10^6 leukocytes, 6.10 x 10^8 erythrocytes, and 5.54 x 10^7 platelets. (**A**) Cytokines detected in the sera that were unaffected by transfusion. Cytokines for which only some of the highest measurements were in range of the standard curve are shown in (**B**) and cytokines whose concentration fell bellow accurate detection levels are shown in (**C**). Significant differences between pre- and post-transfusion are indicated by an asterisk by the name of the mouse strain.
