# Supplementary material for: Immunodeficient mice are better for modeling the transfusion of human blood components than wild-type mice
Source: PLoS One. 2020 Jul 31;15(7):e0237106. doi: 10.1371/journal.pone.0237106 (PMC7394438; doi:10.1371/journal.pone.0237106)
Supplement: S2 Fig — Each transfused group of NSG mice was analyzed using logarithmic (left) and linear (right) regression. Charts are organized by the corresponding Figure in the manuscript. Any additional sub-groups that define the transfused cell population are indicated in the corners of the charts. The results of half-life calculations using the indicated formulas at the top of each chart are shown in Table 2. (PDF) [file pone.0237106.s003.pdf]

Figure 6A

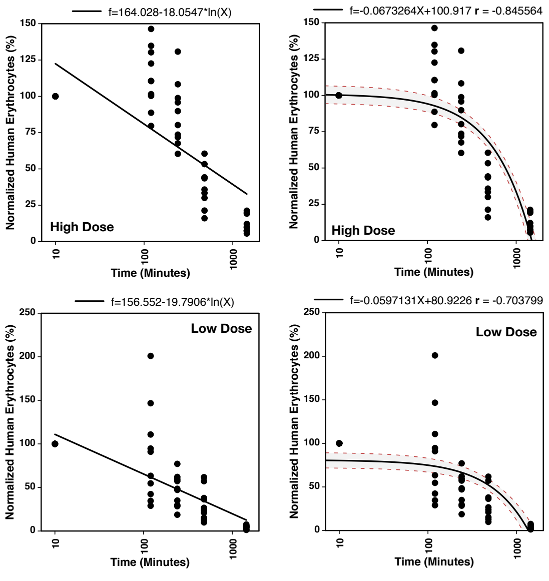

Figure 8C

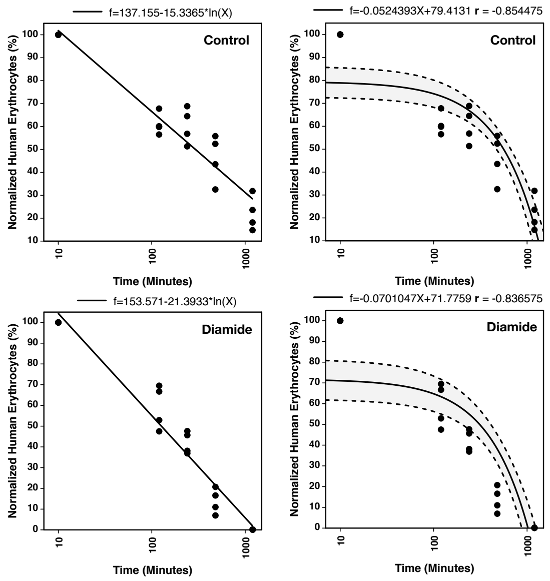

Figure 8A

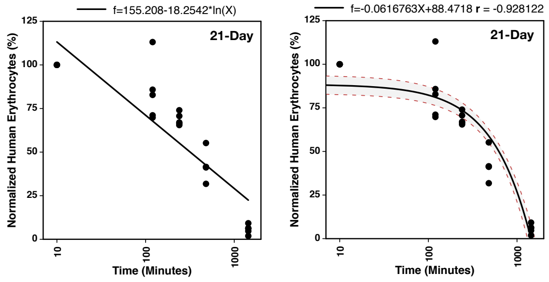

Figure 9D

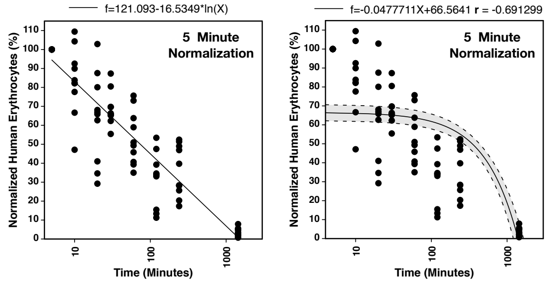

Figure 8B

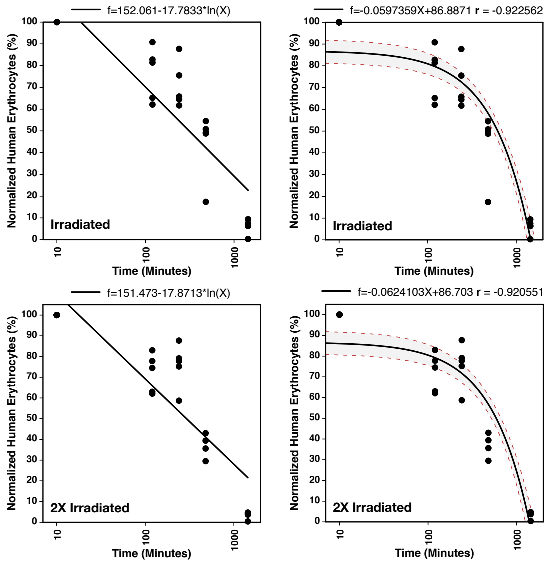

Figure 11A

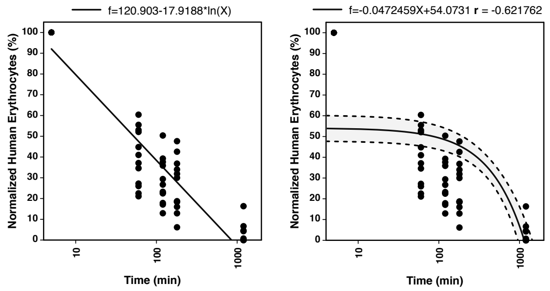

S2 Figure. Half-life determinations for transfused human LR-pRBC. Each transfused group of NSG mice was analyzed using logarithmic (left) and linear (right) regression. Charts are organized by the corresponding Figure in the manuscript. Any additional sub-groups that define the transfused cell population are indicated in the corners of the charts. The results of half-life calculations using the indicated formulas at the top of each chart are shown in Table 2.
